# Paralysie musculaire secondaire à une polymyosite

**DOI:** 10.11604/pamj.2015.20.368.6105

**Published:** 2015-04-15

**Authors:** Meryem Ennafiri, Wafae Elotmani, Almahdi Awab, Rachid El Moussaoui, Ahmed El Hijri, Mustapha Alilou, Abderrahim Azzouzi

**Affiliations:** 1 Service de Réanimation Chirurgicale, Hôpital Avicenne, CHU Ibn Sina, Rabat, Maroc

**Keywords:** Polymyosite, paralysie générale, immunoglobulines intraveineuses, Polymyositis, general paralysis, intravenous immunoglobulin

## Abstract

Les polymyosites sont des maladies inflammatoires des muscles striés, d’étiologie inconnue. Le déficit musculaire, qui se résume généralement à une fatigabilité, évolue de façon bilatérale, symétrique et non sélective avec prédominance sur les muscles proximaux. L'intensité de la faiblesse musculaire est variable d'un sujet à un autre, de la simple gêne fonctionnelle à un état grabataire. Nous rapportons l'observation d'un cas de polymyosite particulièrement sévère avec paralysie musculaire complète, touchant tous les muscles de l'organisme, d’évolution favorable sous immunoglobulines intraveineuses et nous discutons les facteurs favorisant la paralysie musculaire.

## Introduction

Les polymyosites sont des maladies inflammatoires des muscles striés, d’étiologie inconnue. Le déficit musculaire, qui se résume généralement à une fatigabilité, évolue de façon bilatérale, symétrique et non sélective avec prédominance sur les muscles proximaux. L'intensité de la faiblesse musculaire est variable d'un sujet à un autre, de la simple gêne fonctionnelle à un état grabataire [1]. Cette maladie est associée à une mortalité et une morbidité élevée, en particulier liée à une faiblesse musculaire mortelle [2–4]. Nous rapportons l'observation d'une polymyosite particulièrement sévère avec paralysie musculaire complète, touchant tous les muscles de l'organisme, d’évolution favorable sous immunoglobulines intraveineuses.

## Patient et observation

La patiente N.B, âgée de 33 ans, a été hospitalisée pour exploration d'une faiblesse musculaire rhizomélique remontant à un mois et demi avec aggravation progressive. Le diagnostic de polymyosite a été retenu devant l'association de la faiblesse musculaire avec myalgies, l'augmentation des enzymes musculaires (créatinine phospho-kinase (CPK): 622 U/l), un tracé myogène à l’électromyogramme et un aspect évocateur à la biopsie musculaire. 48 heures après le début d'une corticothérapie à base de prédnisolone à 1 mg/kg/j, la patiente a présenté une aggravation de la symptomatologie avec paraplégie flasque d’évolution rapidement progressive puis une gêne respiratoire et des troubles de déglutition nécessitant une intubation trachéale avec ventilation artificielle, réalisée sous propofol (200 mg) et fentanyl (250 µg), sans curare et sans sédation par la suite. Sur les 48 heures suivantes, le déficit musculaire continuait de s'aggraver avec installation d'une tétraplégie flasque, abolition des réflexes ostéo-tendineux et absence de toute réactivité motrice aux stimuli nociceptifs, une paralysie oculomotrice et une mydriase bilatérale aréactive. Par contre, à la stimulation nociceptive, on remarquait un larmoiement, une tachycardie et une augmentation des chiffres tensionnels. La TDM cérébrale était normale. L'examen du LCR n'a pas montré d'anomalie biologique ou cytologique. L'ionogramme sanguin a montré une kaliémie à 4,5 mmol/l, une natrémie à 134 mmol/l, une calcémie à 83 mg/l et une phosphorémie à 38 mg/l. La fonction rénale était normale. Le dosage des enzymes musculaires montrait des CPK à 900 UI/l, une lactico-déshydrogénase à 423 UI/l. Le dosage des anticorps anti-récepteurs de l'acétylcholine était négatif. La patiente est restée sous corticothérapie associée à l'azathioprine (1,5 mg/kg/j) pendant deux semaines sans aucune amélioration clinique, puis instauration d'une cure d'immunoglobulines intraveineuses à raison de 2g/kg sur cinq jours. Une semaine après, soit trois semaines après l'installation de la paralysie générale, une récupération motrice progressive a été notée débutant par les muscles oculomoteurs avec régression de la mydriase, récupération de la mimique du visage et des mouvements distaux des membres supérieurs, puis des muscles respiratoires permettant une respiration spontanée progressive et autorisant le sevrage de la ventilation artificielle après deux mois. Cette amélioration se faisait parallèlement avec baisse des taux d'enzymes musculaires. La déambulation a été possible après trois mois d'hospitalisation. L'interrogatoire à posteriori nous a révélé qu’à aucun moment, la patiente n’était comateuse et qu'elle se souvenait dans le détail de tous les événements par lesquels elle est passée depuis qu'elle était aréactive.

## Discussion

Notre observation offre la particularité de décrire pour la première fois un cas de paralysie générale touchant tous les muscles de l'organisme au cours de la polymyosite. La gravité de cette pathologie est liée essentiellement à la possibilité d'atteinte des muscles pharyngés (10 à 20%) et surtout à l'atteinte respiratoire, présente dans 45% des cas. Une atteinte respiratoire sévère est retrouvée dans 2 à 8% des cas de polymyosite, et représente la première cause de morbidité et de mortalité. Cette atteinte pulmonaire est due à l'inhalation; aux lésions interstitielles, responsables de 30 à 66% de mortalité; ou à l'hypoventilation secondaire à la fatigabilité des muscles respiratoires [5–7]. La paralysie musculaire avec nécessité de ventilation artificielle est rarement décrite. Une association avec d'autres maladies auto-immunes, en particulier avec la myasthénie, peut expliquer la gravité de l'atteinte musculaire dans certains cas. Une paralysie musculaire prolongée a été également décrite après curarisation par du vécuronium. D'autre part, une curarisation significativement prolongée a été remarquée avec du pancuronium, suggérant une prudence à l'utilisation des curares non dépolarisants. Cependant, l'augmentation de la durée de curarisation n'a pas été observée avec le bésilate d'atracurium [8, 9]. Les autres drogues anesthésiques ne semblent pas avoir de répercussion sur la fonction musculaire. Cependant, il est prudent de privilégier les médicaments à durée d'action courte lors de l'anesthésie et la sédation en réanimation.

La paralysie générale dans notre observation n'est pas expliquée par l'association de causes infectieuses, de désordres ioniques ou de curares. D'autre part, les doses cumulatives de corticoïdes reçus sont inférieures aux doses réputées être à l'origine de la myopathie.

À ce jour, les traitements standards des polymyosites incluent la corticothérapie, comme traitement de premier choix, puis les immunosuppresseurs comme traitement de deuxième intension en cas d´effets secondaires ou d’échec de la corticothérapie [10]. Les immunoglobulines intraveineuses est la thérapie recommandée chez les patients atteints de polymyosite réfractaire aux corticoïdes et aux immunosuppresseurs, malgré l'absence d´études contrôlées randomisées [11–13]. Dans notre observation, nous insistons sur l'efficacité des immunoglobulines intraveineuses dans cette forme grave après échec des corticoïdes et des immunosuppresseurs.

## Conclusion

La polymyosite est une maladie inflammatoire responsable de faiblesse musculaire dont l'intensité est variable d'un individu à l'autre. Dans les formes graves nécessitant la ventilation mécanique, les immunoglobulines intraveineuses constituent une alternative efficace au traitement conventionnel par les corticoïdes et les immunosuppresseurs.
